# miR-92b-3p Regulates Cell Cycle and Apoptosis by Targeting *CDKN1C*, Thereby Affecting the Sensitivity of Colorectal Cancer Cells to Chemotherapeutic Drugs

**DOI:** 10.3390/cancers13133323

**Published:** 2021-07-02

**Authors:** Fangqing Zhao, Zhongmin Yang, Xiaofan Gu, Lixing Feng, Mingshi Xu, Xiongwen Zhang

**Affiliations:** Shanghai Engineering Research Center of Molecular Therapeutics and New Drug Development, School of Chemistry and Molecular Engineering, East China Normal University, Shanghai 200062, China; 51184300165@stu.ecnu.edu.cn (F.Z.); 52174300069@stu.ecnu.edu.cn (Z.Y.); 52204300075@stu.ecnu.edu.cn (X.G.); 52164300066@stu.ecnu.edu.cn (L.F.); 51194300165@stu.ecnu.edu.cn (M.X.)

**Keywords:** miR-92b-3p, *CDKN1C*, cell cycle, apoptosis, chemoresistance, colorectal cancer

## Abstract

**Simple Summary:**

Multidrug resistance (MDR) limits the effectiveness of colorectal cancer (CRC) treatment and miRNAs play an important role in drug resistance. To search for miRNA targets that may be involved in the CRC MDR phenotype, this study used small RNAomic screens to analyze the expression profiles of miRNAs in CRC HCT8 cell line and its chemoresistant counterpart HCT8/T cell line. It was found that miR-92b-3p was highly expressed in HCT8/T cells and chemotherapeutic drugs could stimulate CRC cells to up-regulate miR-92b-3p expression and conferred cellular resistance to chemotherapeutic drugs. This study revealed a new mechanism of MDR in CRC, elucidating for the first time the direct link between miR-92b-3p/*CDKN1C* and chemoresistance. In summary, this study suggested that miR-92b-3p could be used as a potential therapeutic target for reversing MDR in chemotherapy and as a candidate biomarker for predicting the efficacy of chemotherapy.

**Abstract:**

Colorectal cancer (CRC) is the third most common malignant tumor in the world and the second leading cause of cancer death. Multidrug resistance (MDR) has become a major obstacle in the clinical treatment of CRC. The clear molecular mechanism of MDR is complex, and miRNAs play an important role in drug resistance. This study used small RNAomic screens to analyze the expression profiles of miRNAs in CRC HCT8 cell line and its chemoresistant counterpart HCT8/T cell line. It was found that miR-92b-3p was highly expressed in HCT8/T cells. Knockdown of miR-92b-3p reversed the resistance of MDR HCT8/T cells to chemotherapeutic drugs in vitro and in vivo. Paclitaxel (PTX, a chemotherapy medication) could stimulate CRC cells to up-regulate miR-92b-3p expression and conferred cellular resistance to chemotherapeutic drugs. In studies on downstream molecules, results suggested that miR-92b-3p directly targeted Cyclin Dependent Kinase Inhibitor 1C (*CDKN1C*, which encodes a cell cycle inhibitor p57Kip2) to inhibit its expression and regulate the sensitivity of CRC cells to chemotherapeutic drugs. Mechanism study revealed that the miR-92b-3p/*CDKN1C* axis exerted a regulatory effect on the sensitivity of CRC cells via the regulation of cell cycle and apoptosis. In conclusion, these findings showed that miR-92b-3p/*CDKN1C* was an important regulator in the development of drug resistance in CRC cells, suggesting its potential application in drug resistance prediction and treatment.

## 1. Introduction

Colorectal cancer (CRC) is the third most common malignant tumor in the world and the second leading cause of cancer death, with only 13% five-year survival rate after tumor spread [1,2,3,4]. Despite the improved efficacy of a multidisciplinary and comprehensive approach based on CRC surgery and standard system chemotherapy, tumors often develop drug resistance as treatment progresses, and are no longer sensitive to chemotherapeutic drugs [5,6,7,8]. At the same time, tumors develop resistance to a variety of drugs with different structures and mechanisms of action, a phenomenon known as multidrug resistance (MDR) in tumors. It is also due to chemotherapy resistance and a lack of predictive biomarkers for standard chemotherapy that survival rates for CRC patients remain poorly improved [6]. Chemotherapy is the standard regimen for patients with this disease, and MDR has become a major obstacle in the clinical treatment of CRC, with nearly 90% of patients developing some degree of MDR during treatment, which prevents clinical treatment from achieving the desired effects, thus causing tumor recurrence and eventual death from cancer [9,10,11]. The development of MDR involves multiple mechanisms, such as increased drug efflux, impaired apoptosis pathway, and altered drug targets, etc. [6,7]. However, the clear molecular mechanism of MDR is complex, and other potential mechanisms and biomarkers remain to be identified.

miRNAs are a class of non-coding RNAs of approximately 22 nucleotides in length encoded by endogenous genes that bind to the 3′ untranslated region (3′ UTR) of the target mRNAs through complementary base-pairing at the post-transcription level, leading to mRNA degradation or translation inhibition, and negatively regulating the expression of target genes [12,13,14]. miRNAs are involved in a variety of biological processes that play important roles in tumor proliferation, migration, invasion, angiogenesis, and drug resistance in the form of oncogenes or tumor suppressor genes [15,16,17,18]. There is increasing evidence that miRNAs, such as miR-302b, miR-23b-3p, miR-199b, and miR-21, play important roles in the MDR of various cancers, including CRC [19,20,21,22]. However, there is still a lack of sufficient studies on the potential role of miRNAs in CRC drug resistance. The relatively non-degradable properties of miRNAs in formalin-fixed and paraffin-embedded materials and in blood make them uniquely advantageous as biomarkers [23,24,25]. To further understand the molecular mechanisms of MDR and to find new biological targets for predicting the effects of chemotherapy, we explored the characteristics and mechanisms of MDR in CRC cell line HCT8 cells and their chemoresistant counterparts HCT8/T cells based on large-scale, unbiased analysis by combining transcriptomics and small RNAomics using high-throughput research techniques. Based on the results of small RNAomics and functional screening, miR-92b-3p was found to be up-regulated in HCT8/T cells compared with HCT8 cells and was selected as a candidate gene for MDR in CRC cells.

As a member of the miR-92b cluster, abnormal expression of miR-92b-3p has been reported in a variety of tumors [26,27,28,29,30,31]. For example, miR-92b-3p expression is up-regulated in glioblastoma and promotes cell proliferation by inhibiting the TGF-β/Smad3/p21 signaling pathway [26]. Interestingly, miR-92b-3p also functions as a tumor suppressor in esophageal cancer, negatively correlating with the presence of local metastasis and good patient prognosis [31]. Although the complex tumor type-specific functions of miR-92b-3p in tumor proliferation, migration and invasion have been studied, the effect of miR-92b-3p on tumor cell drug resistance, especially the role and mechanism of the drug resistance in CRC cells, has not been reported.

This study aims to investigate the role of miR-92b-3p in the development of CRC cells MDR, and to elucidate the mechanism by which it regulates cellular sensitivity to chemotherapeutic drugs through inhibiting the expression of target genes, providing potential targets and biomarkers for future CRC chemotherapy.

## 2. Materials and Methods

### 2.1. Cell Culture

The human CRC cell lines (HCT8, DLD1 and HT29) were purchased from American Type Culture Collection (ATCC, Manassas, VA, USA). The paclitaxel-resistant cell HCT8/T was presented by Professor Jian DING from Shanghai Institute of Materia Medica, Chinese Academy of Sciences. Cells were cultured in RPMI-1640 medium (Hyclone, Logan, UT, USA) containing 10% fetal bovine serum (Gibco, Grand Island, NY, USA) in a humidified incubator with 37 °C, 5% CO_2_. To maintain the MDR phenotype, paclitaxel was added into the HCT8/T cell culture medium, and the final concentration was 1 ug/mL. All the cell lines were regularly identified by morphologic examination and mycoplasma contamination was negative.

### 2.2. Reagents

Paclitaxel (PTX), Etoposide (VP-16) were purchased from Sigma-Aldrich (St. Louis, MO, USA). Doxorubicin (DOX), Vinorelbine (NVB), and Vincristine (VCR) were purchased from MCE (Shanghai, China). Puromycin dihydrochloride (Puro) and Blasticidin S (BSD) were purchased from Beyotime (Shanghai, China). The above is commonly used drugs for chemotherapy. For the in vitro experiment, all drugs were dissolved in dimethyl sulfoxide (DMSO) and stored at −20 °C and diluted to the desired concentrations in Phosphate-buffered saline (PBS) before each experiment. For the in vivo experiment, PTX injection was purchased from Haikou Pharmaceutical Factory (Haikou, China), stored at 4 °C, diluted to the desired concentrations in normal saline before each dosing.

### 2.3. Animals

Female BALB/c nu/nu mice (5–6 weeks old, 16–18g) were purchased from Jihui Laboratory Animal Care (Shanghai, China). The care and experimental protocols for this study complied with Chinese regulations and the Guidelines for the Care and Use of Laboratory Animals drawn up by the National Institutes of Health (United States) and were approved by the Institutional Animal Care and Use Committee of the East China Normal University (m20201207). The mice were maintained on a 12:12 light–dark cycle in a temperature-controlled (21–23 °C), specific pathogen-free (SPF) room, and were provided standard drinking water and diet. All animals were acclimatized for a week before beginning the study.

### 2.4. Small RNA Sequencing

Three replicate RNA samples of HCT8 and HCT8/T were extracted using TRIzol Reagent (Invitrogen, Carlsbad, CA, USA) and used for small RNA sequencing. Sequencing libraries were generated using the TruSeq Small RNA sample prep Kit (Illumina, San Diego, CA, USA) with 1 μg RNA as input material. The normalized small RNA analysis was conducted by Majorbio (Shanghai, China).

### 2.5. Target Prediction

To identify possible molecular mechanisms of action of miR-92b-3p related to MDR, potential targets of miR-92b-3p were predicted using four database algorithms: TargetScan, miRanda, miRDB, PicTar. The screening condition was that the seed region matched at least 7 bases, *p*-value < 0.05, and four algorithms were all predicted.

### 2.6. Cell Transfection

For the overexpression of miR-92b-3p, inserting the miR-92b-3p sequence into a Puro-resistant lentiviral vector to construct a plasmid that overexpressed miR-92b-3p, with an empty vector used as a control. For the inhibition of miR-92b-3p expression, the short hairpin RNA (shRNA)-mediated knockdown of miR-92b-3p was performed. The shRNA sequence was as follows: 5′-TATTGCACTCGTCCCGGACTACCTTCCTGTCAGAGGAGGCCGGGACGAGTGCAATA-3′. The shRNA was inserted into a BSD-resistant lentiviral vector to construct a plasmid that inhibited miR-92b-3p, with an empty vector used as a control. 293/T cells were cultured to 50–60% confluence and then transfected with the miR-92b-3p overexpression plasmid, the miR-92b-3p knockdown plasmid, and respective controls using Lipofectamine 3000 (Invitrogen, Carlsbad, CA, USA) according to the manufacturer’s instructions. After 48 h, lentivirus particles infected HCT8 and HCT8/T cells were collected, and then selected by Puro or BSD to establish the HCT8 cells stably overexpressing miR-92b-3p and HCT8/T cells stably knockdown miR-92b-3p for further experiments. miR-92b-3p overexpressing HCT8 cells and its control cells were named HCT8 miR-92b-3p and HCT8 NC, respectively. miR-92b-3p knockdown HCT8/T cells and its control cells were named HCT8/T shmiR-92b-3p and HCT8/T shNC, respectively.

For the inhibition and overexpression of Cyclin Dependent Kinase Inhibitor 1C (*CDKN1C*, which encodes a cell cycle inhibitor p57Kip2), HCT8, HCT8/T, HCT8 miR-92b-3p, HCT8/T shmiR-92b-3p were cultured to 50–60% confluence and then transfected with the *CDKN1C* overexpression vector, *CDKN1C* siRNAs (purchased from RiboBio, Guangzhou, China), and respective controls using Lipofectamine 3000 or Lipofectamine RNAiMAX (Invitrogen, Carlsbad, CA, USA) according to the manufacturer’s instructions. *CDKN1C* overexpressing HCT8/T cells and its control cells were named HCT8/T *CDKN1C* and HCT8/T Vector, respectively. *CDKN1C* knockdown HCT8 cells and its control cells were named HCT8 si*CDKN1C* and HCT8 siNC, respectively. *CDKN1C* overexpressing HCT8 miR-92b-3p cells and its control cells were named HCT8 miR-92b-3p+*CDKN1C* and HCT8 miR-92b-3p+Vector, respectively. *CDKN1C* knockdown HCT8/T shmiR-92b-3p cells and its control cells were named HCT8/T shmiR-92b-3p+si*CDKN1C* and HCT8/T shmiR-92b-3p+siNC, respectively.

### 2.7. RNA Extraction and qRT-PCR

Total RNA was extracted from the tissues and cells using TRIzol reagent (Takara, Shiga, Japan). After synthesizing cDNAs with the M-MLV reverse transcriptase (Promega, WI, USA), the expression levels of miR-92b-3p and its target genes were analyzed using SYBR Premix EX TaqII Kit (TaKaRa, Tokyo, Japan) and ran in triplicate on CFX96 Touch Deep Well Real-Time PCR System (Bio-Rad, CA, USA). The fold change of miRNA or mRNA expression was calculated according to the 2^−∆∆Ct^ method and presented as relative to the U6 miRNA and *GAPDH* mRNA levels. All primer sequences used for qRT-PCR were from Genewiz (Suzhou, China) and listed in Table S1.

### 2.8. Cell Proliferation Assay

Cell proliferation was evaluated using the Cell Counting Kit 8 (CCK8; Dojindo, Japan) according to the manufacturer’s protocol. CRC cells were seeded into 96-well plates at 2000 cells/well and 100 μL RPMI-1640 medium containing 10% CCK8 was added to each well at the indicated time points. Cells were further incubated for 2 h in a humidified incubator with 37 °C, 5% CO_2_. The absorbance was measured at 450 nm with a SpectraMax M5 microplate reader (Molecular Devices, Sunnyvale, CA, USA).

### 2.9. Drug Sensitivity Assay

The survival ratio of cells was analyzed using the CCK8 according to the manufacturer’s protocol. After transfection as described previously, CRC cells were seeded into 96-well plates at 3000 cells/well, allowed to attach overnight, and treated with chemotherapeutic drugs at gradient concentrations for 72 h. Then, the CCK8 assay was performed. The survival rate (%) was calculated as follows: [(OD_experiment_ − OD_blank_)/(OD_control_ − OD_blank_)] × 100% and the IC_50_ values were calculated based on a non-linear regression analysis.

### 2.10. Cell Cycle Assay

The distribution of cells in the G1, S, and G2/M cell cycle phases was determined by flow cytometry. After transfection as described previously, CRC cells were harvested, washed with PBS, fixed with ice-cold 70% ethanol overnight at 4 °C, incubated with RNase at 37 °C for 15 min, and then stained with propidium iodide (PI) for an additional 15 min. The cell cycle was measured using CytoFLEX S flow cytometer (Beckman, Brea, CA, USA), and DNA histograms were analyzed using FlowJo software.

### 2.11. Apoptosis Assay

The apoptosis percentage was measured by an Annexin-V-FITC/PI apoptosis detection kit (Vazyme, Nanjing, China). After 24 h of transfection as described previously, the cells were treated with the different concentrations of PTX for 24 h. According to the manufacturer’s instructions, 1 × 10^6^ cells were washed twice with PBS, resuspended in 1× Binding Buffer, and then stained with 5 μL Annexin V-FITC and 5 μL PI on ice for 15 min, followed by adding 400 μL 1× Binding Buffer. The apoptosis percentage was analyzed using CytoFLEX S flow cytometer (Beckman, Brea, CA, USA) and FlowJo software.

### 2.12. Dual Luciferase Reporter Assay

The human *CDKN1C* 3′ UTR fragment containing the predicted miR-92b-3p binding site was amplified and cloned into a psiCHECK-2 vector (Promega, Madison, WI, USA), to generate the plasmid psiCHECK-2-*CDKN1C*-3′ UTR-WT (*CDKN1C* WT). psiCHECK-2-*CDKN1C*-3′ UTR-MUT (*CDKN1C* MUT) was generated from *CDKN1C* WT plasmid by mutating the putative binding site of miR-92b-3p in the *CDKN1C* 3′ UTR. 293/T cells were co-transfected with *CDKN1C* WT, *CDKN1C* MUT and NC, or miR-92b-3p plasmids using Lipofectamine3000. After transfection for 48 h, firefly luciferase and Renilla luciferase activities were analyzed using the Dual-Luciferase Reporter Assay System (Promega, Madison, WI, USA) according to the manufacturer’s instructions, and the firefly luciferase activity was normalized to Renilla luciferase activity results.

### 2.13. Western Blotting Analysis

Cells were lysed in RIPA buffer plus a phosphatase protease inhibitor. Tissues were lysed in RIPA buffer with a tissue lyser (Qiagen, Valencia, CA, USA). The protein concentration was measured by BSA assay. Equal amounts of protein samples were subjected to 10% SDS-PAGE gel electrophoresis and transferred to nitrocellulose membrane. The membrane was blocked in 5% non-fat milk in PBS containing 0.1% Tween 20 (TPBS) for 2 h at room temperature and then incubated with primary antibody diluted in TPBS containing 5% Bovine Serum Albumin (BSA) at 4 °C overnight. After washing three times for 10 min each time on the next day, and then incubated with secondary antibody in TPBS containing 5% non-fat milk for 1 h at room temperature, the protein signals were captured using ECL Chemiluminescent Kit (Thermo Fisher, Waltham, MA, USA) and Amersham Imager 600 (GE, Boston, MA, USA). The primary antibodies used were as follows: p57Kip2 (rabbit anti-p57Kip2 polyclonal antibody, 1:1000, Cell Signaling Technology, Danvers, MA, USA), GAPDH (GAPDH-HRP, 1:5000, Santa Cruz Biotechnology, CA, USA). HRP-conjugated goat anti-mouse and anti-rabbit secondary antibodies were from Multi Sciences (1:5000, Hangzhou, China). The protein level was defined as the ratio of the experimental group to the control group.

### 2.14. Nude Mice Xenograft Tumor Assay

Nude mice (*n* = 6) were inoculated subcutaneously at right and left flanks with HCT8/T shNC and HCT8/T shmiR-92b-3p cells (1 × 10^6^). After 1–2 weeks, well-grown xenografts were cut into 1.5 mm^3^ fragments and transplanted subcutaneously into the right flank of nude mice. When tumors reached an average volume of 70–120 mm^3^, mice were randomly assigned into three groups (*n* = 7) and received various treatments for 21 days: (i) vehicle (intraperitoneal injection, q3d); (ii) NVB (4 mg/kg, intraperitoneal injection, q3d); (iii) PTX (20 mg/kg, intraperitoneal injection, q3d). The volume of the tumors and the body weight of the mice were measured individually every two days. Tumor volume was calculated as follows: V = (width^2^ × length)/2. Relative tumor volume (RTV) was calculated as V_t_/V_0_, where t represents the days of treatment, V_0_ and V_t_ were the volumes before and after treatment, respectively. The T/C (%) value and the tumor growth inhibition rate (TGI %) were used to determine the in vivo anticancer activity of each treatment group. T/C (%) was calculated as (TRTV/CRTV) × 100%, where TRTV and CRTV represented the RTV of the treatment group and the vehicle group, respectively. The tumor growth inhibition rate was calculated as TGI (%) = (1 − (average tumor volume of the treatment group on the end day − average tumor volume of the treatment group on the first day)/(average tumor volume of the vehicle group on the end day − average tumor volume of the vehicle group on the first day)) × 100%.

### 2.15. Statistical Analysis

Data were presented as the mean ± SD. Two-tailed Student’s t-test was used for comparisons between two groups. All analyses were performed using GraphPad Prism 8.0 software. *p* < 0.05 was considered to be statistically significant and results were presented as * *p* < 0.05; ** *p* < 0.01; *** *p* < 0.001.

## 3. Results

### 3.1. miR-92b-3p Is Highly Expressed in HCT8/T Cells and Induced by PTX

**Figure 1:**
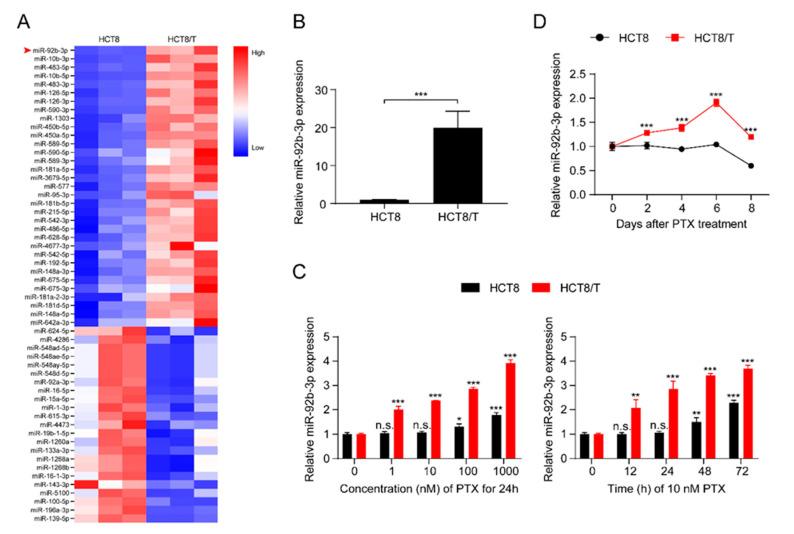
miR-92b-3p is highly expressed in HCT8/T cells and induced by PTX. (**A**) Heatmap of miRNAs expression profiling in HCT8 and HCT8/T cells measured. The red-white-blue color scale represents the normalized expression value of miRNAs: low expression (blue), medium expression (white), and high expression (red). (**B**) qRT-PCR analysis of relative expression levels of miR-92b-3p in HCT8 and HCT8/T cells. *** *p* < 0.001. (**C**) qRT-PCR analysis of relative expression levels of miR-92b-3p in HCT8 and HCT8/T cells treated with different concentrations of PTX for 24 h and 10 nM PTX for different time. versus cells without PTX treatment, respectively, n.s. *p* > 0.05; * *p* < 0.05; ** *p* < 0.01; *** *p* < 0.001. (**D**) qRT-PCR analysis of relative expression levels of miR-92b-3p in HCT8 and HCT8/T cells over time after treatment with 10 nM PTX for 72 h. versus HCT8 cells, *** *p* < 0.001. All experiments above were repeated three or more times independently and values are shown as mean ± SD.

MDR HCT8/T cells are more resistant to multiple chemotherapeutic drugs (PTX, DOX, NVB, VCR, VP-16) than their parental HCT8 cells (Table S2). The development of MDR is multi-factorial and may be influenced by unknown biological mechanisms. To further understand the role of miRNAs in the development of MDR, the expression profiles of miRNAs in the pair of cell lines, HCT8 and HCT8/T, were analyzed and evaluated using a small RNAomics approach, and 220 differentially expressed miRNAs were detected. To further narrow the scope of miRNAs and improve the reliability of the results, the screening criteria were further defined as: transcripts per kilobase of exon model per million mapped reads (TPM) > 10, fold change (FC) > 2 or < 0.5, and a total of 56 differentially expressed candidate miRNAs were finally obtained (Figure 1A). miR-92b-3p with the largest FC was selected as the candidate gene for MDR in CRC cells by combining the results of small RNA histology and functional screening. Quantitative real-time polymerase chain reaction (qRT-PCR) results showed that, consistent with the histological results, miR-92b-3p was significantly up-regulated in HCT8/T cells compared with HCT8 cells (Figure 1B). The results indicated that miR-92b-3p might be an important candidate biomarker of MDR. Since HCT8/T cells were induced by long-term PTX on the basis of HCT8 cells, we further investigated the effect of PTX on miR-92b-3p expression in CRC cells. HCT8 and HCT8/T cells were treated with different concentrations (1 nM, 10 nM, 100 nM, 1000 nM) of PTX for 24 h, or with 10 nM PTX for different time periods (12 h, 24 h, 48 h, 72 h). qRT-PCR analysis showed that miR-92b-3p expression was significantly up-regulated after PTX treatment in a concentration-dependent and time-dependent manner, while the response of HCT8/T cells to PTX was significantly stronger than that of HCT8 cells (Figure 1C). The same up-regulated trend was also observed in other CRC cells DLD1 and HT29 (Figures S1A and S2A). To assess the long-term effect of PTX on the expression of miR-92b-3p, HCT8 and HCT8/T cells were treated with 10 nM of PTX for 72 h, then the drug was withdrawn and the expression level of miR-92b-3p was measured on days 0, 2, 4, 6, and 8, respectively. The results showed that miR-92b-3p expression was progressively up-regulated in both HCT8 and HCT8/T cells. HCT8/T cells responded more strongly to PTX, and their miR-92b-3p expression levels peaked on day six and then gradually decreased, while HCT8 cells responded weakly to PTX and gradually decreased after a slight increase (Figure 1D). These findings indicated that the expression of miR-92b-3p in CRC cells could be induced by PTX in a concentration-dependent and time-dependent manner, and miR-92b-3p levels were continuously elevated (in HCT8/T cells) or remained basically unchanged (in HCT8 cells) after PTX withdrawal. It is speculated that miR-92b-3p expression might be up-regulated by the chemotherapeutic agent during the development of HCT8/T MDR, stably and highly expressed in HCT8/T cells after long-term stimulation, and more rapidly and strongly induced by the chemotherapeutic agent to protect the cells from the chemotherapeutic drugs when the cells were stimulated by chemotherapy again.

### 3.2. miR-92b-3p Maintains the Cell Viability in a Chemotherapy Setting

**Figure 2:**
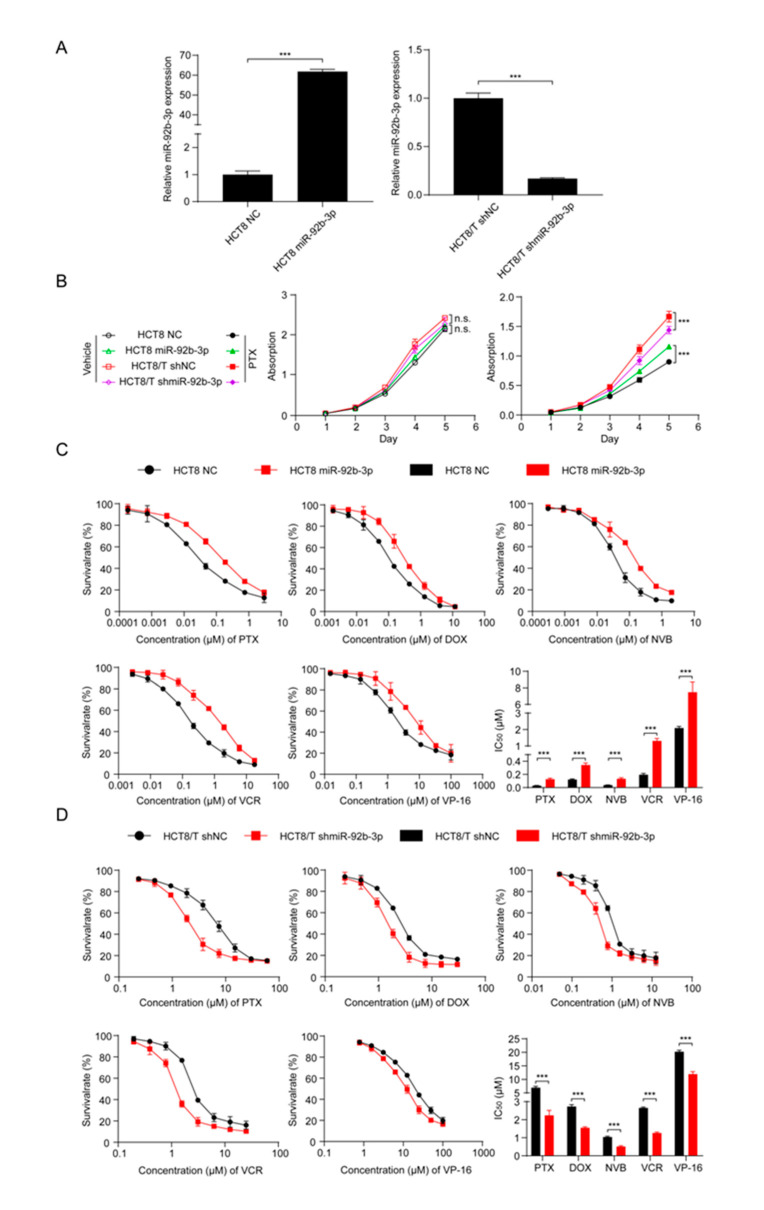
miR-92b-3p maintains the cell viability and promotes the chemoresistance of HCT8 and HCT8/T cells to chemotherapeutic drugs. (**A**) qRT-PCR analysis of the efficiency of miR-92b-3p overexpression in HCT8 cells and knockdown in HCT8/T cells. (**B**) miR-92b-3p maintains the cell viability in the presence of PTX. Cells were treated with or without 10 nM PTX for different time and then subjected to CCK8 assay. (**C**) Overexpression of miR-92b-3p desensitized HCT8 cells to chemotherapeutic drugs, and (**D**) Knockdown of miR-92b-3p resensitized HCT8/T cells to chemotherapeutic agents. Cells were treated with drugs at gradient concentrations for 72 h, then cell survival curves and IC_50_ values of multiple chemotherapeutic drugs were determined by CCK8 assay. All experiments above were repeated three or more times independently and values are shown as mean ± SD. *** *p* < 0.001.

To investigate the potential biological function of miR-92b-3p in CRC cells, miR-92b-3p was forced to be expressed in parental HCT8 cells, and the stable knockdown of its expression in HCT8/T cells was achieved using shRNA targeting miR-92b-3p. qRT-PCR confirmed the efficiency of miR-92b-3p overexpression and knockdown (Figure 2A). In order to determine the effect of miR-92b-3p on cell proliferation and its role on cell viability in the presence of PTX, cell proliferation was examined under co-culture conditions without PTX and with 10 nM PTX using the CCK8 assay. The results showed that miR-92b-3p only weakly promoted cell proliferation in the absence of PTX. In contrast, in the presence of PTX, miR-92b-3p played an important role in maintaining cell viability in the chemotherapy setting. The proliferation rates of HCT8/T shNC and HCT8 miR-92b-3p cells in the presence of PTX were significantly higher than those of the corresponding HCT8/T shmiR-92b-3p and HCT8 NC cells (Figure 2B). In conclusion, these results suggested that miR-92b-3p maintained the proliferation of CRC cells in a chemotherapy setting.

### 3.3. Overexpression of miR-92b-3p Desensitizes HCT8 Cells to Chemotherapeutic Drugs and Knockdown of miR-92b-3p Resensitizes HCT8/T Cells to Chemotherapeutic Drugs

To further investigate the relationship between miR-92b-3p and chemotherapy resistance, the CCK8 method was used to detect changes in drug sensitivity caused by miR-92b-3p overexpression and knockdown. miR-92b-3p overexpression significantly enhanced the resistance of HCT8 cells to PTX, DOX, NVB, VCR, and VP-16 (Figure 2C), while miR-92b-3p knockdown restored the sensitivity of HCT8/T cells to PTX and other chemotherapeutics (Figure 2D). Compared with control HCT8 NC cells, the sensitivity of HCT8 miR-92b-3p cells to PTX and other chemotherapeutics was reduced by 2.81–6.72 folds; on the contrary, the sensitivity of HCT8/T shmiR-92b-3p cells to chemotherapeutics was significantly increased by 1.70–3.09 folds (Table 1). These results further suggested that miR-92b-3p promoted the MDR level of HCT8/T cells. In summary, miR-92b-3p could maintain cell viability in a chemotherapy setting and promote the resistance of CRC cells to chemotherapeutic drugs.

### 3.4. miR-92b-3p Directly Targets CDKN1C and Suppresses Its Expression

**Figure 3:**
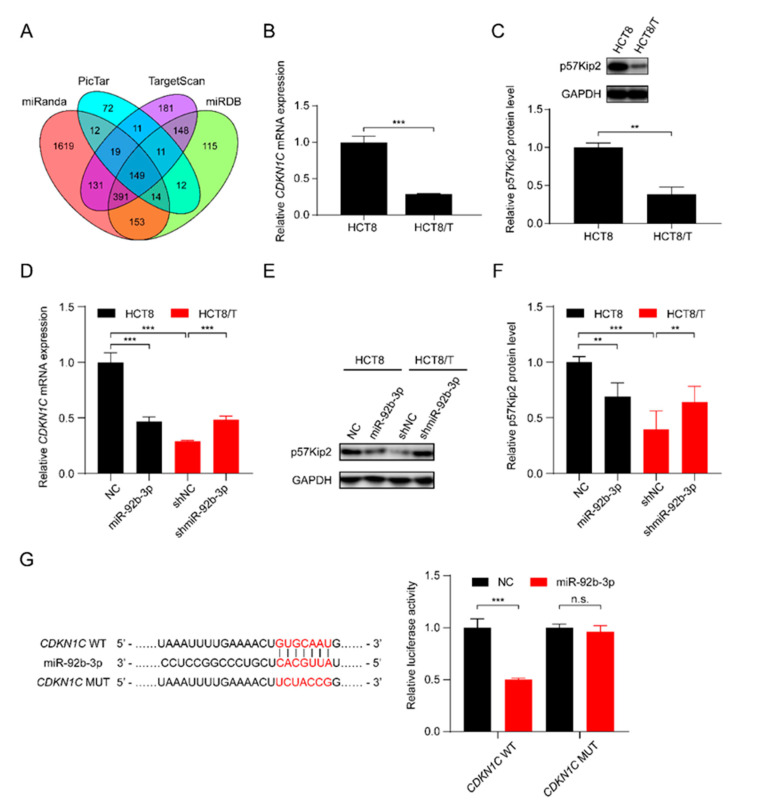
miR-92b-3p directly targets *CDKN1C* and suppresses its expression. (**A**) The potential target of miR-92b-3p by bioinformatics analysis (**B**,**C**) The p57Kip2 expression levels in HCT8 and HCT8/T cells by qRT-PCR and western blotting analysis. Detailed information about Western Blot can be found at Supplementary Materials. (**D**–**F**) The p57Kip2 expression levels in miR-92b-3p overexpressing HCT8 cells and miR-92b-3p knockdown HCT8/T cells by qRT-PCR and western blotting analysis. Detailed information about Western Blot can be found at Supplementary Materials. (**G**) Validation of the direct target of miR-92b-3p by dual luciferase reporter assay. Firefly luciferase and Renilla luciferase activities in 293/T cells after co-transfection with NC or miR-92b-3p and wild-type or mutant 3′ UTR of *CDKN1C* by dual luciferase reporter assay. All experiments above were repeated three or more times independently and values are shown as mean ± SD. n.s. *p* > 0.05; ** *p* < 0.01; *** *p* < 0.001.

In order to further elucidate the potential molecular mechanisms involved in the regulatory effect of miR-92b-3p on CRC cells, the bioinformatics algorithms TargetScan, miRanda, miRDB, and PicTar were used to predict the possible targets of miR-92b-3p, and finally *CDKN1C*, which was predicted as a target by all of the four tools, was selected as a potential target gene of miR-92b-3p (Figure 3A). qRT-PCR and western blotting results showed that *CDKN1C* was down-regulated in HCT8/T cells compared with HCT8 cells (Figure 3B,C). Furthermore, overexpression of miR-92b-3p suppressed the levels of mRNA and protein expression of *CDKN1C* in HCT8 cells, whereas downregulation of miR-92b-3p resulted in the opposite changes of *CDKN1C* expression in HCT8/T cells (Figure 3D–F). The above results suggested that miR-92b-3p negatively regulated the expression of *CDKN1C*. To determine whether miR-92b-3p directly regulated *CDKN1C* expression, the wild-type and mutated 3′ UTR sequences of *CDKN1C* predicted to interact with miR-92b-3p were created and cloned into the psiCHECK-2 luciferase reporter gene vector (Figure 3G). Dual luciferase assays showed that miR-92b-3p inhibited the luciferase activity of wild-type *CDKN1C* 3′ UTR sequence, but had no effect on the mutant *CDKN1C* 3′ UTR sequence (Figure 3G). These results suggest that *CDKN1C* is a direct target of miR-92b-3p. Taken together, miR-92b-3p directly targets *CDKN1C* and inhibits its expression.

### 3.5. CDKN1C Silencing Reduces the Sensitivity of HCT8 Cells to Chemotherapeutic Drugs and Overexpression of CDKN1C Restores the Sensitivity of HCT8/T Cells to Chemotherapeutic Drugs

**Figure 4:**
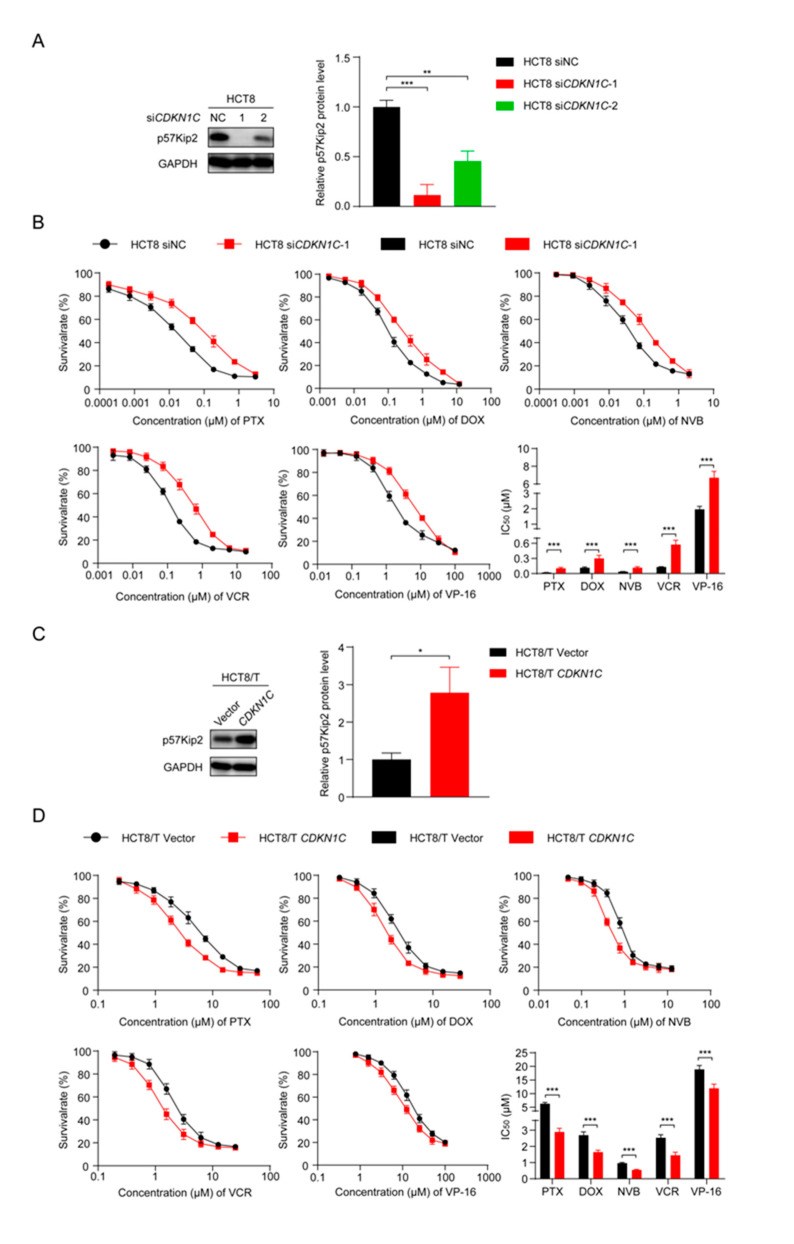
*CDKN1C* increases the sensitivity of HCT8 and HCT8/T cells to chemotherapeutic drugs. (**A**) Western blotting analysis of p57Kip2 expression levels in HCT8 cells treated with si*CDKN1C* for 48 h, and (**C**) in HCT8/T cells treated with *CDKN1C* for 48 h. Detailed information about Western Blot can be found at Supplementary Materials. (**B**) Silencing of *CDKN1C* desensitized HCT8 cells to chemotherapeutic drugs, and (**D**) Overexpression of *CDKN1C* resensitized HCT8/T cells to chemotherapeutic agents. Cells were treated with drugs at gradient concentrations for 72 h after transfection with *CDKN1C* or si*CDKN1C*-1 for 24 h, cell survival curves and IC_50_ values of multiple chemotherapeutic drugs were measured by CCK8 assay. All experiments above were repeated three or more times independently and values are shown as mean ± SD. * *p* < 0.05; ** *p* < 0.01; *** *p* < 0.001.

To further explore the role of *CDKN1C* in the MDR of CRC cells, two small interfering siRNAs targeting *CDKN1C* (si*CDKN1C*-1 and si*CDKN1C*-2) were synthesized and transfected into HCT8 cells. Western blotting confirmed their inhibition, and the silencing effect of si*CDKN1C*-1 was better (Figure 4A). Therefore, si*CDKN1C*-1 was used in subsequent experiments to inhibit the expression of *CDKN1C*. The changes in drug sensitivity caused by *CDKN1C* silencing were detected using CCK8 assay and, as expected, *CDKN1C* silencing reduced the sensitivity of HCT8 cells to PTX and other chemotherapeutics (Figure 4B). In contrast, overexpression of *CDKN1C* restored the sensitivity of HCT8/T cells to PTX and other chemotherapeutics (Figure 4C,D). Compared with the effect of miR-92b-3p expression on drug sensitivity, the effect of *CDKN1C* expression on drug sensitivity was relatively weak: *CDKN1C* silencing reduced the sensitivity of HCT8 cells to chemotherapeutics only by 2.63–5.5 folds, while *CDKN1C* overexpression increased the sensitivity of chemotherapeutics by 1.58–2.17 folds (Table 2). It is suggested that *CDKN1C* played an important role in CRC MDR and was a direct functional target gene of miR-92b-3p. miR-92b-3p regulated the sensitivity of CRC cells to chemotherapeutics through *CDKN1C* or at least partially through *CDKN1C* in vitro.

### 3.6. miR-92b-3p Regulates the Sensitivity of HCT8 and HCT8/T Cells to Chemotherapeutic Drugs by Targeting CDKN1C

**Figure 5:**
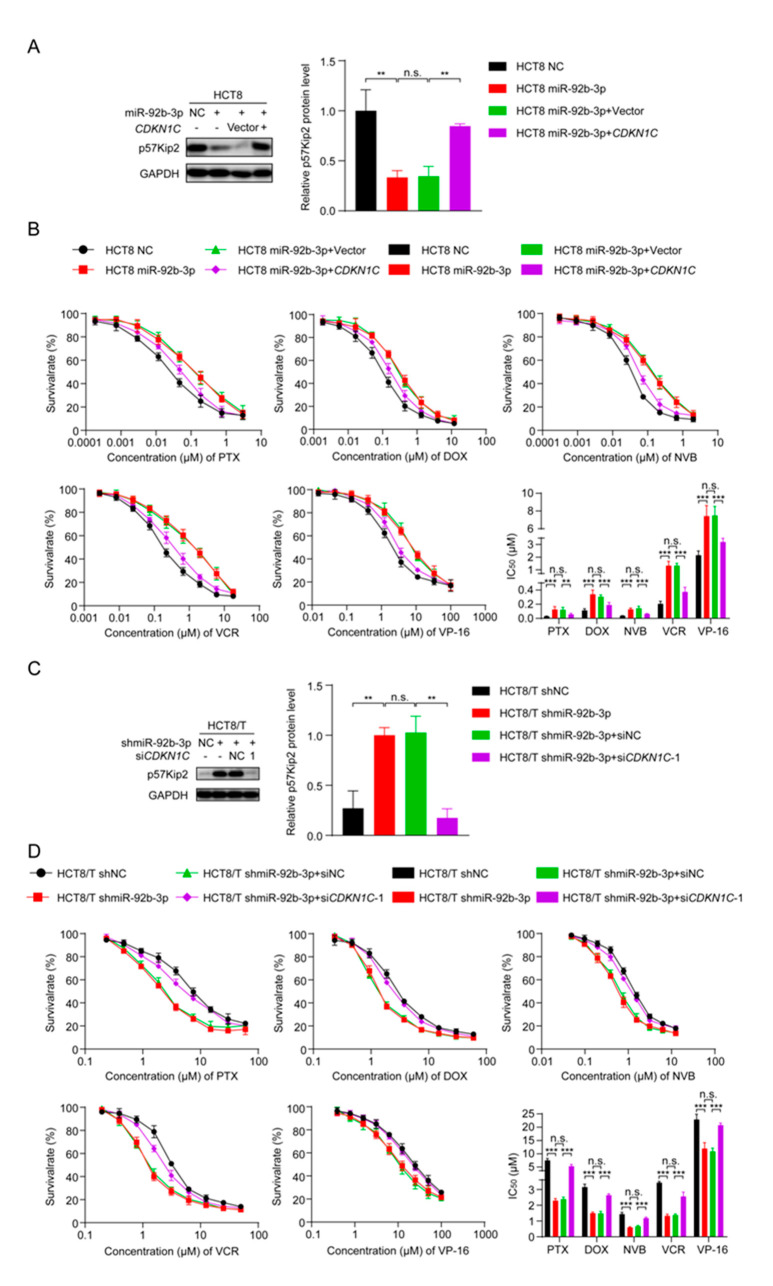
miR-92b-3p regulates the sensitivity of HCT8 and HCT8/T cells to chemotherapeutic drugs by targeting *CDKN1C*. (**A**) Western blotting analysis of the p57Kip2 expression levels in HCT8 cells transfected with miR-92b-3p or co-transfected with miR-92b-3p and *CDKN1C*, and (**C**) in HCT8/T cells transfected with shmiR-92b-3p or co-transfected with shmiR-92b-3p and si*CDKN1C-1*. Detailed information about Western Blot can be found at Supplementary Materials. (**B**) Overexpression of *CDKN1C* attenuates the desensitization effect of miR-92b-3p on HCT8 cells to chemotherapeutic drugs, and (**D**) Silencing of *CDKN1C* reverses the sensitization effect of miR-92b-3p knockdown on HCT8/T cells to chemotherapeutic drugs. Cells were treated with drugs at gradient concentrations for 72 h after transfection of miR-92b-3p or shmiR-92b-3p or co-transfection with miR-92b-3p and *CDKN1C* or co-transfection with shmiR-92b-3p and si*CDKN1C-1* for 24 h, cell survival curves and IC_50_ values of multiple chemotherapeutic drugs were measured by CCK8 assay. All experiments above were repeated three or more times independently and values are shown as mean ± SD. n.s. *p* > 0.05; ** *p* < 0.01; *** *p* < 0.001.

To clarify whether miR-92b-3p regulated the sensitivity of CRC cells to chemotherapeutic drugs by inhibiting *CDKN1C*, changes in cell sensitivity to chemotherapeutic drugs were examined using the CCK8 assay after overexpression of miR-92b-3p and *CDKN1C*. Interestingly, the co-expression of *CDKN1C* and miR-92b-3p attenuated the inhibitory effect of miR-92b-3p on *CDKN1C* expression (Figure 5A) and the desensitization effect on HCT8 cells (Figure 5B and Table 3). In contrast, co-silencing of miR-92b-3p and *CDKN1C* reversed the sensitizing effect of miR-92b-3p silencing on HCT8/T cells (Figure 5C,D and Table 4). Taking PTX as an example, drug resistance of HCT8/T cells was significantly reduced after miR-92b-3p silencing (IC_50_ decreased from 7.52 ± 0.68 μM to 2.29 ± 0.13 μM), and cellular drug resistance was restored (IC_50_ from 2.38 ± 0.13 μM to 5.33 ± 0.62 μM) after *CDKN1C* co-silencing. In addition, miR-92b-3p also showed the same effect on other CRC cell lines DLD1 and HT29 cells, overexpression of miR-92b-3p reduced the expression of *CDKN1C* and the sensitivity to chemotherapy. The co-expression of *CDKN1C* and miR-92b-3p attenuated the inhibitory effect of miR-92b-3p on *CDKN1C* expression and the desensitization effect on both DLD1 and HT29 cells (Figures S1 and S2, Tables S3 and S4). In summary, these data suggested that miR-92b-3p could promote CRC cell resistance to chemotherapeutics by regulating *CDKN1C*.

### 3.7. The miR-92b-3p/CDKN1C Axis Mediates the Sensitivity of HCT8 and HCT8/T Cells to Chemotherapeutic Drugs through Regulating Cell Cycle and Apoptosis

**Figure 6:**
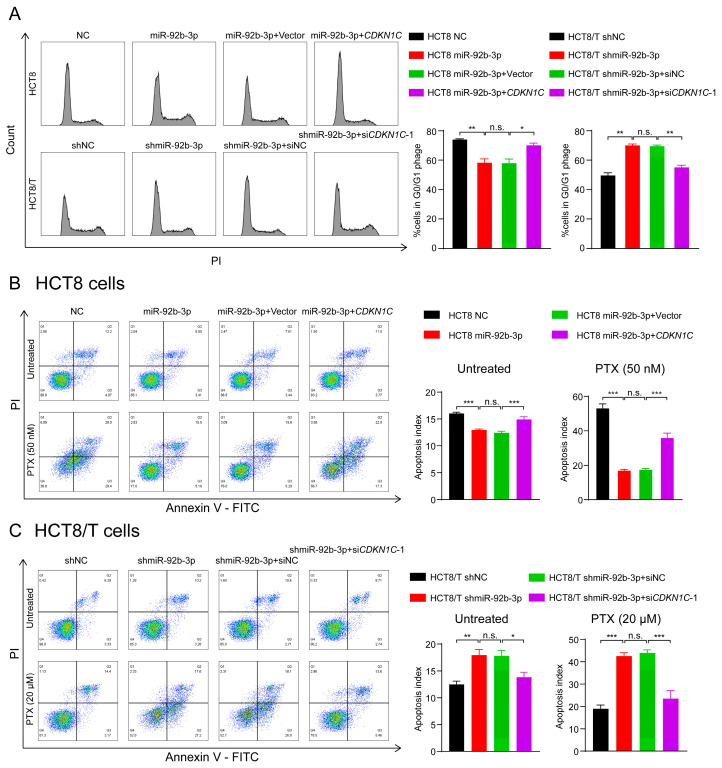
The miR-92b-3p/*CDKN1C* axis mediates the sensitivity of HCT8 and HCT8/T cells to chemotherapeutic drugs through regulating cell cycle and apoptosis. (**A**) miR-92b-3p reduces G1 phase arrest in HCT8 and HCT8/T cells by targeting *CDKN1C*. Cell cycle distribution of HCT8 cells transfected with miR-92b-3p or co-transfected with miR-92b-3p and *CDKN1C*, and HCT8/T cells transfected with shmiR-92b-3p or co-transfected with shmiR-92b-3p and si*CDKN1C-1* by flow cytometry. (**B**,**C**) miR-92b-3p contributes to reduction of cell apoptosis in the chemotherapy setting by targeting *CDKN1C*. 24 h after transfection, HCT8 cells were treated with PTX (50 nM) for another 24 h, HCT8/T cells were treated with PTX (20 μM) for another 24 h. Apoptosis detection was measured by an Annexin-V apoptosis detection kit. All experiments above were repeated three or more times independently and values are shown as mean ± SD. n.s. *p* > 0.05; * *p* < 0.05; ** *p* < 0.01; *** *p* < 0.001.

P57Kip2 protein encoded by *CDKN1C* is a member of the Cip/Kip family of cyclin-dependent kinase inhibitors that acts as a cell cycle regulatory protein by binding to and inhibiting the activity of cyclin A/CDK2 and cyclin E/CDK2 complexes, thereby leading to cellular block in G1 phase and preventing DNA synthesis [32,33]. Thus, HCT8 cells may exhibit a stronger blocking effect in G1 phase. Consistent with the predicted results, there was an increased number of HCT8 cells in G1 phase and decreased numbers of HCT8 cells in S and G2/M phases compared to HCT8/T cells (Figure 6A). To further determine the effects of miR-92b-3p and *CDKN1C* on cell cycle distribution, cells were subjected to cell cycle detection using flow cytometry. The results showed that both miR-92b-3p overexpression and *CDKN1C* silencing could reduce G1 phase arrest in HCT8 cells, while miR-92b-3p and *CDKN1C* co-expression weakened the effect of miR-92b-3p overexpression; in contrast, miR-92b-3p knockdown and *CDKN1C* overexpression both triggered G1 phase block in HCT8/T cells, while miR-92b-3p and *CDKN1C* co-silencing reversed the triggering effect of knocking down miR-92b-3p on G1 phase block (Figure 6A). These results confirmed that miR-92b-3p regulated cell cycle arrest by targeting *CDKN1C*.

The previous results showed that the chemotherapy setting has an important influence on the role of miR-92b-3p. Therefore, the following hypothesis can be proposed: miR-92b-3p may contribute to reduction of cell apoptosis in the chemotherapy setting, and this effect may be achieved by targeting *CDKN1C*. To test this hypothesis, flow cytometry was used to compare the changes in apoptosis of cells treated with PTX before and after. The results showed that miR-92b-3p and *CDKN1C* had little effect on cell apoptosis without PTX treatment, but for PTX-treated cells, miR-92b-3p could significantly reduce PTX-induced cell apoptosis, while *CDKN1C* could significantly promote PTX-induced cell apoptosis. A further finding was that overexpression of *CDKN1C* attenuated the inhibitory effect of miR-92b-3p on HCT8 cell apoptosis under chemotherapy, while siRNA targeting *CDKN1C* reversed the pro-apoptotic effect of miR-92b-3p knockdown on HCT8/T cell apoptosis in the chemotherapy setting (Figure 6B,C), suggesting that the regulatory effect of miR-92b-3p on apoptosis in the chemotherapy setting was *CDKN1C*-mediated. The results supported the above speculation that miR-92b-3p could reduce cell apoptosis in the chemotherapy setting through the specific regulation of *CDKN1C*. In summary, the miR-92b-3p/*CDKN1C* axis affected the sensitivity of cells to chemotherapeutic drugs by regulating cell cycle and apoptosis.

### 3.8. miR-92b-3p Suppresses the Sensitivity of HCT8/T Xenograft Tumors to Chemotherapeutic Drugs

**Figure 7:**
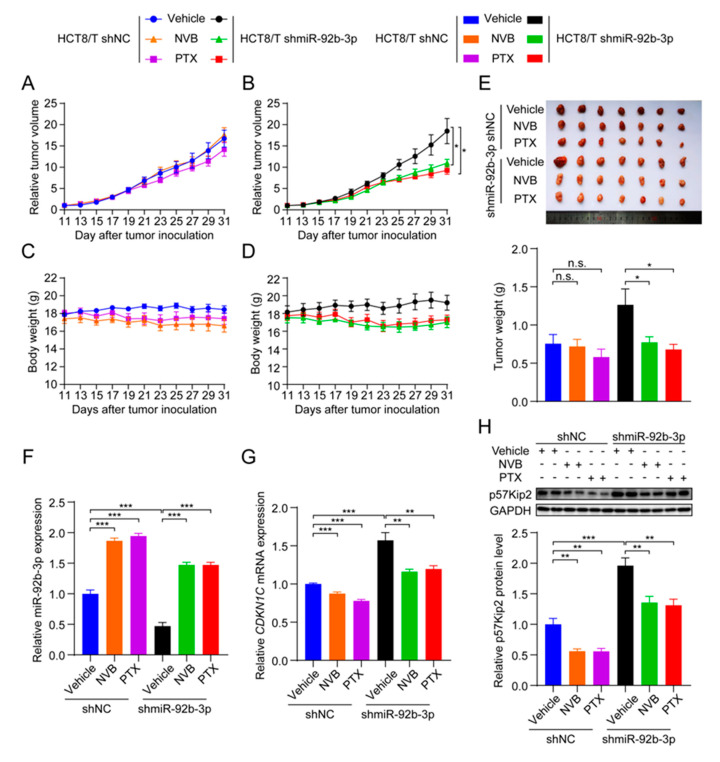
miR-92b-3p suppresses the sensitivity of HCT8/T xenograft tumors to chemotherapeutic drugs. Nude mice bearing CRC xenografts with HCT8/T control and miR-92b-3p knockdown cells were administered NVB (4 mg/kg) or PTX (20 mg/kg) by intraperitoneal injection every three days for 21 days and vehicle group mice received equal volume solvent. Tumor volume and body weight were recorded every two days. (**A**–**D**) The change in relative tumor volume and body weight over time. (**E**) The image and weight of the xenograft tumors at the end of treatments. (**F**) Relative miR-92b-3p expression levels in xenograft tumors evaluated by qRT-PCR. (**G**,**H**) The *CDKN1C* expression levels in xenograft tumors by qRT-PCR and western blotting analysis. Detailed information about Western Blot can be found at Supplementary Materials. n.s. *p* > 0.05; * *p* < 0.05; ** *p* < 0.01; *** *p* < 0.001.

To test whether miR-92b-3p affected the sensitivity of CRC cells to chemotherapeutic drugs in vivo, HCT8/T shmiR-92b-3p cells and control cells were used to establish nude mouse xenograft models. Consistent with the results of in vitro studies, HCT8/T shmiR-92b-3p cells showed no significant change in tumor growth rate compared with control cells. Chemotherapeutic drug treatment significantly inhibited the growth of HCT8/T shmiR-92b-3p xenograft tumors, with 44.32% and 53.23% inhibition rates after NVB and PTX treatment, respectively, compared with only −2.18% and 15.83% inhibition rates after chemotherapy in control HCT8/T shNC xenograft tumors (Figure 7A,B and Table 5). The chemotherapeutic drug carrier and chemotherapeutic drugs had no significant effect on mice body weight (Figure 7C,D). There was no significant difference in tumor volume and tumor weight between the chemotherapeutic drug treatment and vector control groups of HCT8/T shNC xenograft tumors, whereas the tumor volume and tumor weight of the HCT8/T shmiR-92b-3p xenograft tumors were significantly lower compared with the vector control group (Figure 7E). In addition, qRT-PCR and western blotting results showed that miR-92b-3p and *CDKN1C* had similar expression profiles in xenograft tumor models as in CRC cell models (Figure 7F–H). These data suggested that knockdown of miR-92b-3p up-regulated *CDKN1C* expression, significantly enhanced the response of HCT8/T xenografts to chemotherapeutics and reversed drug resistance.

## 4. Discussion

An increasing number of studies have shown that miRNAs play a key role in the development of tumor MDR [34]. From the present study, it was found that miR-92b-3p level was significantly elevated in MDR HCT8/T cells and could be up-regulated by PTX induction. Furthermore, miR-92b-3p overexpression could maintain cell viability in the chemotherapy setting and resistance to chemotherapeutics, whereas miR-92b-3p knockdown had the opposite effects in vitro and in vivo. As a member of the miR-92b cluster, miR-92b-3p differential expression has been reported in a variety of cancers, including glioma, non-small-cell lung cancer, and pancreatic cancer [26,27,28,29,30]. Interestingly, the expression and role of miR-92b-3p were not consistent in different cancers. For example, some studies proposed that miR-92b-3p could promote the proliferation of glioblastoma cells by inhibiting the TGF-β/Smad3/p21 signaling pathway and inhibited cell apoptosis by targeting the PTEN/Akt signaling pathway [26,27]. In addition to its carcinogenic effect in brain tumor tissues, the anticancer effect of miR-92b-3p had also been reported [29,30]. For example, miR-92b-3p had low expression in lung cancer cells and inhibited the proliferation and invasion of lung cancer cells by targeting EZH2 [29]. It also inhibited the proliferation, migration and invasion of cancer cells in pancreatic cancer [30]. The above contradictory results were mainly due to the fact that each miRNA could potentially affect and control more than 100 target genes through binding to the 3′ UTR of the target gene mRNA, and positively or negatively regulated the cancer network in a cellular environment-dependent manner [35]. Therefore, different cancer types showed seemingly different functions of miR-92b-3p, reflecting the intrinsic complexity and diversity of tumor biology. On the other hand, the biological effects of miR-92b-3p were largely influenced by the cellular environment and the specific genetic background of the tumor type, which reminded us to proceed with caution when using miRNA therapies clinically.

Although the role of miR-92b-3p in cell proliferation, migration, and invasion has been demonstrated, the association between miR-92b-3p and MDR, especially with CRC MDR, has not been reported. In this study, the results of both in vitro and in vivo experiments confirmed the key role of miR-92b-3p in CRC drug resistance. These results suggested that chemotherapeutic drugs could stimulate CRC cells to up-regulate miR-92b-3p expression and conferred cellular resistance to chemotherapeutic drugs. This mechanism explained, at least partially, the role and mechanism of miR-92b-3p involvement in the development of chemotherapeutic drug resistance in CRC cells. In addition, this study further confirmed that miR-92b-3p was not only an important candidate biomarker for clinical chemotherapy outcome prediction, but also a possible new target for clinical treatment of CRC. These results improved our understanding of MDR-related miRNAs in CRC and supported the development of miR-92b-3p-based CRC therapeutics. To the best of our knowledge, this study identified miR-92b-3p as a key regulator of CRC drug sensitivity for the first time.

In our studies on downstream molecules of miR-92b-3p, double luciferase reporter gene analysis and western blotting analysis showed that miR-92b-3p directly targeted and inhibited the expression of *CDKN1C*, which encodes p57Kip2 protein. *CDKN1C* overexpression significantly reversed the MDR in HCT8/T cells, while co-silencing of miR-92b-3p and *CDKN1C* reversed the sensitizing effect of miR-92b-3p silencing on HCT8/T cells. It was already known that *CDKN1C* was an imprinted gene (maternally expressed gene) located on human chromosome 11p15.5, and that the expression was epigenetically regulated and usually considered as a tumor suppressor gene involved in tumorigenesis, with reduced expression or inactivation in a variety of tumors, such as ovarian, lung, and bladder cancer [36]. Moreover, the mutation/inactivation of *CDKN1C* in the cancer-susceptible Beckwith–Wiedemann syndrome also implied its importance in tumor suppression [37]. Now, it has been found that *CDKN1C*-encoded p57Kip2 is also involved in the regulation of various cellular processes such as transcription, differentiation, and migration through its PAPA repeat sequence and carboxy-terminal structural domain [32,33]. However, whether *CDKN1C* is also involved in the MDR of tumor cells, and its role and mechanism in the MDR of CRC cells remain unclear. The results of this study suggested that miR-92b-3p regulated drug sensitivity of CRC cells by targeting *CDKN1C*, whereas knockdown of miR-92b-3p up-regulated *CDKN1C* expression, significantly reversed drug resistance in vivo and in vitro. In this study, the direct relationship between miR-92b-3p/*CDKN1C* and chemoresistance was elucidated for the first time, and revealed a new mechanism of MDR in CRC.

On further investigation of the intrinsic biological mechanism, the results showed that the miR-92b-3p/*CDKN1C* axis could regulate cell cycle arrest. It has been reported that *CDKN1C* is a member of the Cip/Kip family of cyclin-dependent kinase inhibitors, banded tightly to the complex formed by CDK2, CDK3, and CDK4, and also had some inhibitory effect on the complex formed by CDK1 and CDK6 [32]. It was a negative regulator of cell proliferation, which mainly caused cell cycle arrest in G1 phase [33]. Consistent with these reports, the results of this study showed that CRC cells with high *CDKN1C* expression were blocked in G1 phase. Meanwhile, miR-92b-3p decreased the mRNA and protein levels of *CDKN1C*, thereby affecting G1 phase arrest and cell cycle distribution. It has previously been found that cell cycle arrest could slow down DNA synthesis and activate different cell death pathways, such as apoptosis, mitotic catastrophe and necrosis. Chemotherapeutic drugs for CRC clinical treatment play an anti-tumor role mainly by interfering with DNA and RNA synthesis [38,39,40]. In conclusion, miR-92b-3p regulated cell cycle by targeting *CDKN1C*, thereby affecting the sensitivity of CRC cells.

Further studies revealed that *CDKN1C* could significantly promote cell apoptosis in a chemotherapy setting, but had little effect on apoptosis in the non-chemotherapy environment. Based on this, we speculated that the presence or absence of chemotherapy was a key factor in the effect of p57Kip2 on apoptosis. Rossi and Antonangeli demonstrated in previous studies that *CDKN1C* encoded protein p57Kip2 had the ability to regulate cell apoptosis through different mechanisms, and its pro- and anti-apoptotic effects were influenced by the environment of cells and the stress of cells [41]. In this study, the results suggested that p57Kip2 enhanced chemotherapeutic drug-induced cell apoptosis and exerted a pro-apoptotic effect in the chemotherapy environment, while reduced p57Kip2 levels protected cells from damage and reduced apoptosis in the chemotherapy setting. These data suggested that the regulatory effect of *CDKN1C* on apoptosis in a chemotherapy setting might be a key mechanism by which *CDKN1C* regulated the sensitivity of CRC cells to chemotherapeutic drugs. Apoptosis plays a central role in regulating the homeostasis of normal cells and tissues, and most chemotherapeutic drugs currently in clinical use exert anti-tumor effects primarily through stimulating apoptosis mechanism [41]. P57Kip2 was shown by Samuelsson to enhance the apoptotic effects of staurosporine on HeLa cells by promoting the cell-intrinsic apoptotic pathway [42,43]. In conclusion, in the absence of chemotherapeutic drugs, p57Kip2 only caused cell G1 phase arrest and had little effect on cell apoptosis. When cells were stimulated by chemotherapeutics, p57Kip2 could promote the mitochondrial apoptosis pathway, thereby enhancing drug-induced apoptosis and improving cell sensitivity to chemotherapeutic drugs.

In summary, miR-92b-3p regulated cell cycle and apoptosis by targeting *CDKN1C*, affecting cell sensitivity to chemotherapeutic drugs. The different roles of *CDKN1C* under different conditions reflects the complex and coordinated network that exist during cancer development, with its mechanism of action undergoing a series of changes due to environmental influences. More understanding of the regulatory role of *CDKN1C* in apoptosis, especially in the chemotherapy setting, may provide more clarity on the mechanisms of drug resistance in tumor cells and bring new insights to clinical treatment.

## 5. Conclusions

**Figure 8:**
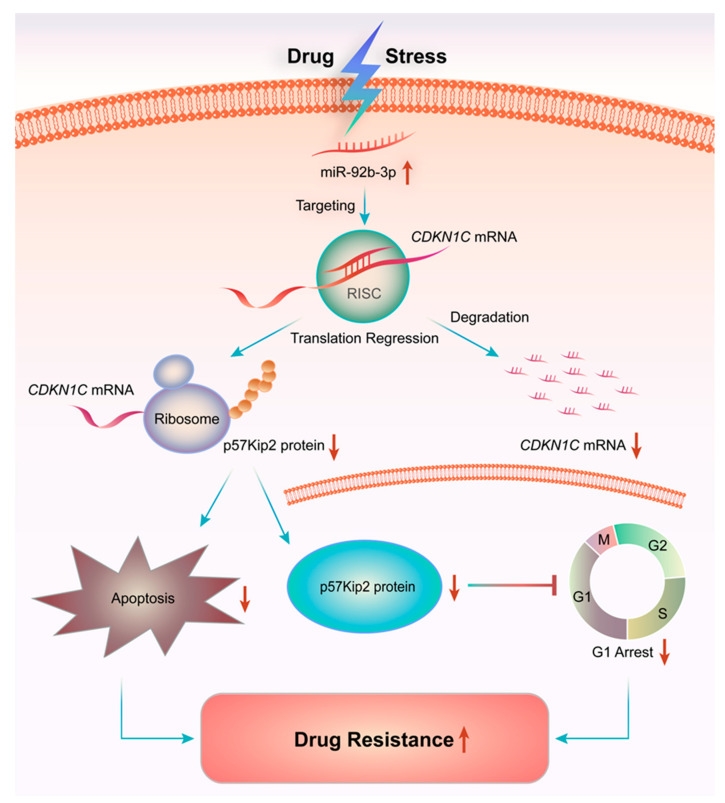
miR-92b-3p regulates chemotherapeutic drug resistance by targeting *CDKN1C*. Chemotherapeutic agents such as PTX could up-regulate the expression of miR-92b-3p, thereby inhibiting the expression of target gene *CDKN1C*, reducing cell cycle G1 arrest and apoptosis in chemotherapy, which promotes the increase of cell tolerance to chemotherapy drugs and multidrug resistance.

Overall, the present study showed that miR-92b-3p was highly expressed in HCT8/T MDR cells and was up-regulated by PTX. Furthermore, in vivo and in vitro experiments showed that knocking down miR-92b-3p restored the sensitivity of HCT8/T cells to chemotherapeutic drugs by targeting *CDKN1C*. The possible mechanism of miR-92b-3p was to induce cell G1 arrest by targeting *CDKN1C*, promote the mitochondrial apoptosis pathway, and enhance the induction of apoptosis by chemotherapeutic drugs. This study revealed a new mechanism of MDR in CRC, elucidating for the first time the direct link between miR-92b-3p/*CDKN1C* and chemoresistance (Figure 8). Although further studies are needed to clearly define the role of miR-92b-3p-mediated molecular mechanisms in controlling the development of MDR, this work suggested that miR-92b-3p might play an important role in the development of MDR, which indicated that miR-92b-3p could be used as a potential therapeutic target for reversing MDR in chemotherapy and as a candidate biomarker for predicting the efficacy of chemotherapy.

## Figures and Tables

**Table 1:** The effect of miR-92b-3p on sensitivity of HCT8 and HCT8/T cells to chemotherapeutic drugs.

Drugs	IC_50_ (Mean ± SD, μM)		Fold Resistance	IC_50_ (Mean ± SD, μM)		Fold Reversal
	HCT8 NC	HCT8 miR-92b-3p		HCT8/T shNC	HCT8/T shmiR-92b-3p	
PTX	0.0286 ± 0.0044	0.1279 ± 0.0154 ***	4.47	6.92 ± 0.50	2.24 ± 0.28 ***	3.09
DOX	0.1217 ± 0.0093	0.3418 ± 0.0318 ***	2.81	2.73 ± 0.10	1.55 ± 0.06 ***	1.76
NVB	0.0377 ± 0.0024	0.1331 ± 0.0158 ***	3.53	1.04 ± 0.04	0.51 ± 0.05 ***	2.04
VCR	0.1949 ± 0.0207	1.3090 ± 0.1442 ***	6.72	2.65 ± 0.05	1.26 ± 0.05 ***	2.10
VP-16	2.0910 ± 0.0995	7.4720 ± 1.2260 ***	3.57	20.22 ± 0.54	11.92 ± 0.93 ***	1.70

Cell survival curves of multiple chemotherapeutic agents were measured by CCK8 assay after 72 h treatment with agents (in gradient concentrations) and the IC_50_ values were calculated based on a non-linear regression analysis. The fold resistance was calculated as the IC_50_ value of HCT8 miR-92b-3p cells divided by the IC_50_ value of HCT8 NC cells. The fold reversal was calculated as the IC_50_ value of HCT8/T shNC cells divided by the IC_50_ value of HCT8/T shmiR-92b-3p cells. All experiments above were repeated three or more times independently and values are shown as mean ± SD. versus control cells, respectively. *** *p* < 0.001.

**Table 2:** The effect of *CDKN1C* on sensitivity of HCT8 and HCT8/T cells to chemotherapeutic drugs.

Drugs	IC_50_ (Mean ± SD, μM)		Fold Resistance	IC_50_ (Mean ± SD, μM)		Fold Reversal
	HCT8 siNC	HCT8 si*CDKN1C*-1		HCT8/T Vector	HCT8/T *CDKN1C*	
PTX	0.0188 ± 0.0027	0.1034 ± 0.0182 ***	5.50	6.27 ± 0.52	2.89 ± 0.23 ***	2.17
DOX	0.1142 ± 0.0164	0.2999 ± 0.0625 ***	2.63	2.69 ± 0.21	1.64 ± 0.12 ***	1.64
NVB	0.0386 ± 0.0035	0.1168 ± 0.0192 ***	3.03	0.96 ± 0.05	0.54 ± 0.04 ***	1.78
VCR	0.1300 ± 0.0061	0.5744 ± 0.0864 ***	4.42	2.53 ± 0.19	1.45 ± 0.20 ***	1.74
VP-16	1.9590 ± 0.1971	6.7030 ± 0.7310 ***	3.42	18.89 ± 1.40	11.96 ± 1.53 ***	1.58

Cell survival curves of multiple chemotherapeutic agents were measured by CCK8 assay after 72 h treatment with agents (in gradient concentrations) and the IC_50_ values were calculated based on a non-linear regression analysis. The fold resistance was calculated as the IC_50_ value of HCT8 si*CDKN1C*-1 cells divided by the IC_50_ value of HCT8 siNC cells. The fold reversal was calculated as the IC_50_ value of HCT8/T Vector cells divided by the IC_50_ value of HCT8/T *CDKN1C* cells. All experiments above were repeated three or more times independently and values are shown as mean ± SD. versus control cells, respectively. *** *p* < 0.001.

**Table 3:** Overexpression of *CDKN1C* reverses the effect of miR-92b-3p overexpression on sensitivity of HCT8 cells to chemotherapeutic drugs. Cell survival curves of multiple chemotherapeutic agents were measured by CCK8 assay after 72 h treatment with agents (in gradient concentrations) and the IC_50_ values were calculated based on a non-linear regression analysis. All experiments above were repeated three or more times independently and values are shown as mean ± SD. versus control cells, respectively. ** *p* < 0.01; *** *p* < 0.001.

Drugs	IC_50_ (Mean ± SD, μM)			
	HCT8 NC	HCT8 miR-92b-3p	HCT8 miR-92b-3p+Vector	HCT8 miR-92b-3p+*CDKN1C*
PTX	0.0282 ± 0.0052	0.1252 ± 0.0421 ***	0.1213 ± 0.0353	0.0566 ± 0.0140 **
DOX	0.1130 ± 0.0227	0.3400 ± 0.0606 ***	0.3058 ± 0.0255	0.1896 ± 0.0347 ***
NVB	0.0362 ± 0.0029	0.1268 ± 0.0193 ***	0.1417 ± 0.0297	0.0618 ± 0.0062 ***
VCR	0.2046 ± 0.0374	1.3190 ± 0.3520 ***	1.3310 ± 0.1793	0.3749 ± 0.0643 ***
VP-16	2.1490 ± 0.3348	7.4280 ± 1.1860 ***	7.5020 ± 1.0160	3.2020 ± 0.2855 ***

**Table 4:** Silencing of *CDKN1C* reverses the effect of miR-92b-3p knockdown on drug resistance of HCT8/T cells to chemotherapeutic drugs. Cell survival curves of multiple chemotherapeutic agents were measured by CCK8 assay after 72 h treatment with agents (in gradient concentrations) and the IC_50_ values were calculated based on a non-linear regression analysis. All experiments above were repeated three or more times independently and values are shown as mean ± SD. versus control cells, respectively. *** *p* < 0.001.

Drugs	IC_50_ (Mean ± SD, μM)			
	HCT8/T shNC	HCT8/T shmiR-92b-3p	HCT8/T shmiR-92b-3p+siNC	HCT8/T shmiR-92b-3p+si*CDKN1C*-1
PTX	7.52 ± 0.68	2.29 ± 0.13 ***	2.38 ± 0.13	5.33 ± 0.62 ***
DOX	3.15 ± 0.18	1.50 ± 0.07 ***	1.48 ± 0.13	2.63 ± 0.10 ***
NVB	1.44 ± 0.11	0.59 ± 0.05 ***	0.65 ± 0.04	1.18 ± 0.06 ***
VCR	3.43 ± 0.08	1.33 ± 0.10 ***	1.38 ± 0.07	2.56 ± 0.27 ***
VP-16	22.88 ± 1.97	11.94 ± 2.23 ***	10.91 ± 1.17	20.77 ± 0.78 ***

**Table 5:** Knockdown of miR-92b-3p increases the sensitivity of HCT8/T xenograft tumors to chemotherapeutic drugs. The change of body weight, tumor volume, tumor weight, and RTV over time after HCT8/T shNC and shmiR-92b-3p cells inoculation. versus vehicle group, respectively. * *p* < 0.05.

Cell Lines	Group	Body Weight (g)		Tumor Volume (mm^3^)		Tumor Weight (g)	RTV	T/C	TGI (%)
		D11	D31	D11	D31				
HCT8/T shNC	Vehicle	17.87 ± 0.20	18.44 ± 0.43	75.42 ± 8.31	1235.14 ± 182.40	0.76 ± 0.12	16.72 ± 2.00	-	-
	NVB	17.40 ± 0.53	16.60 ± 0.71	74.80 ± 8.34	1259.77 ± 100.69	0.72 ± 0.09	17.65 ± 1.65	1.05	−2.18
	PTX	18.03 ± 0.36	17.43 ± 0.66	74.54 ± 8.36	1050.67 ± 163.01	0.58 ± 0.10	14.17 ± 1.61	0.85	15.83
HCT8/T shmiR-92b-3p	Vehicle	18.16 ± 0.71	19.21 ± 0.84	114.00 ± 17.20	1999.24 ± 336.23	1.26 ± 0.21	18.48 ± 2.96	-	-
	NVB	17.53 ± 0.59	17.00 ± 0.60	113.20 ± 15.06	1162.95 ± 70.84 *	0.77 ± 0.07 *	10.96 ± 0.89 *	0.65	44.32
	PTX	17.74 ± 0.53	17.29 ± 0.53	111.30 ± 16.92	992.94 ± 152.68 *	0.68 ± 0.07 *	9.29 ± 0.93 *	0.55	53.23

## Data Availability

The data presented in this study are available on request from the corresponding author. The data are not publicly available due to privacy.

## Supplementary Materials

The following are available online at https://www.mdpi.com/article/10.3390/cancers13133323/s1, Figure S1: miR-92b-3p regulates the sensitivity of DLD1 cells to chemotherapeutic drugs by targeting *CDKN1C*, Figure S2: miR-92b-3p regulates the sensitivity of HT29 cells to chemotherapeutic drugs by targeting *CDKN1C*. Table S1: Primer sequences used for qRT-PCR, Table S2: The sensitivity of HCT8 and HCT8/T cells to chemotherapeutic drugs, Table S3: Overexpression of *CDKN1C* reverses the effect of miR-92b-3p overexpression on sensitivity of DLD1 cells to chemotherapeutic drugs, Table S4: Overexpression of *CDKN1C* reverses the effect of miR-92b-3p overexpression on sensitivity of HT29 cells to chemotherapeutic drugs. Origional Western Blot figures.
