# Supplementary material for: miR-92b-3p Regulates Cell Cycle and Apoptosis by Targeting CDKN1C, Thereby Affecting the Sensitivity of Colorectal Cancer Cells to Chemotherapeutic Drugs
Source: Cancers (Basel). 2021 Jul 2;13(13):3323. doi: 10.3390/cancers13133323 (PMC8268555; doi:10.3390/cancers13133323)
Supplement: Supplementary file 1 [file cancers-13-03323-s001.zip › cancers-1231163-supplementary.pdf]

cancers

MDPI

Supplementary Materials

miR-92b-3p Regulates Cell Cycle and Apoptosis by Targeting *CDKN1C*, Thereby Affecting the Sensitivity of Colorectal Cancer Cells to Chemotherapeutic Drugs

Fangqing Zhao, Zhongmin Yang, Xiaofan Gu, Lixing Feng, Mingshi Xu and Xiongwen Zhang

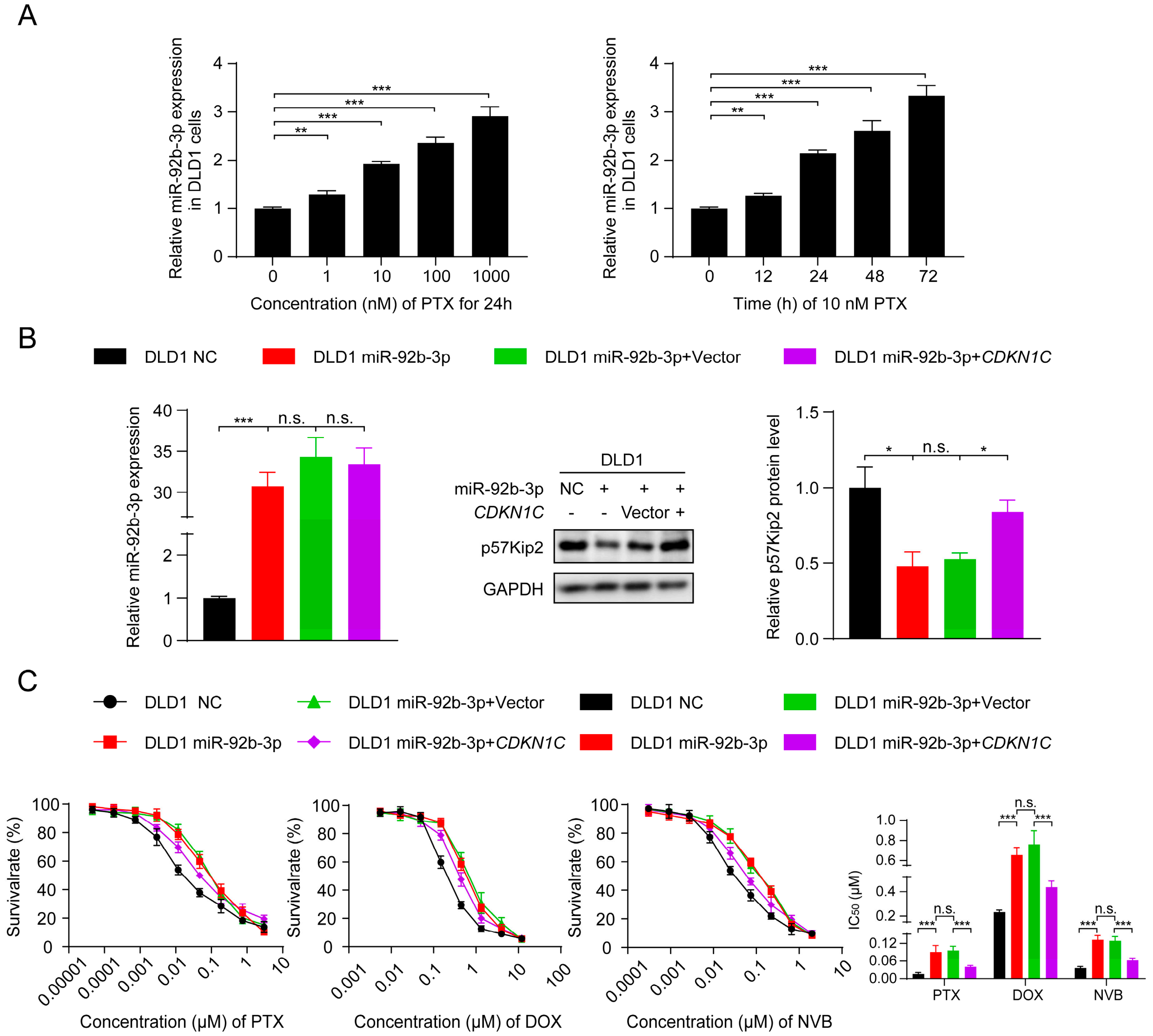

Figure S1. miR-92b-3p regulates the sensitivity of DLD1 cells to chemotherapeutic drugs by targeting *CDKN1C*. (**A**) qRT-PCR analysis of relative expression levels of miR-92b-3p in DLD1 cells treated with different concentrations of PTX for 24 h and 10 nM PTX for different time. (**B**) Western blotting analysis of the p57Kip2 expression levels in DLD1 cells transfected with miR-92b-3p or co-transfected with miR-92b-3p and *CDKN1C*. (**C**) Overexpression of *CDKN1C* attenuates the desensitizes effect of miR-92b-3p on DLD1 cells to chemotherapeutic drugs. Cells were treated with drugs at gradient concentrations for 72h after transfection of miR-92b-3p or cotransfection with miR-92b-3p and *CDKN1C* for 24 h, cell survival curves and IC_{50} values of multiple chemotherapeutic drugs were measured by CCK8 assay. All experiments above were repeated three or more times independently and values are shown as mean ± SD. *$p < 0.05$; **$p < 0.01$; ***$p < 0.001$.

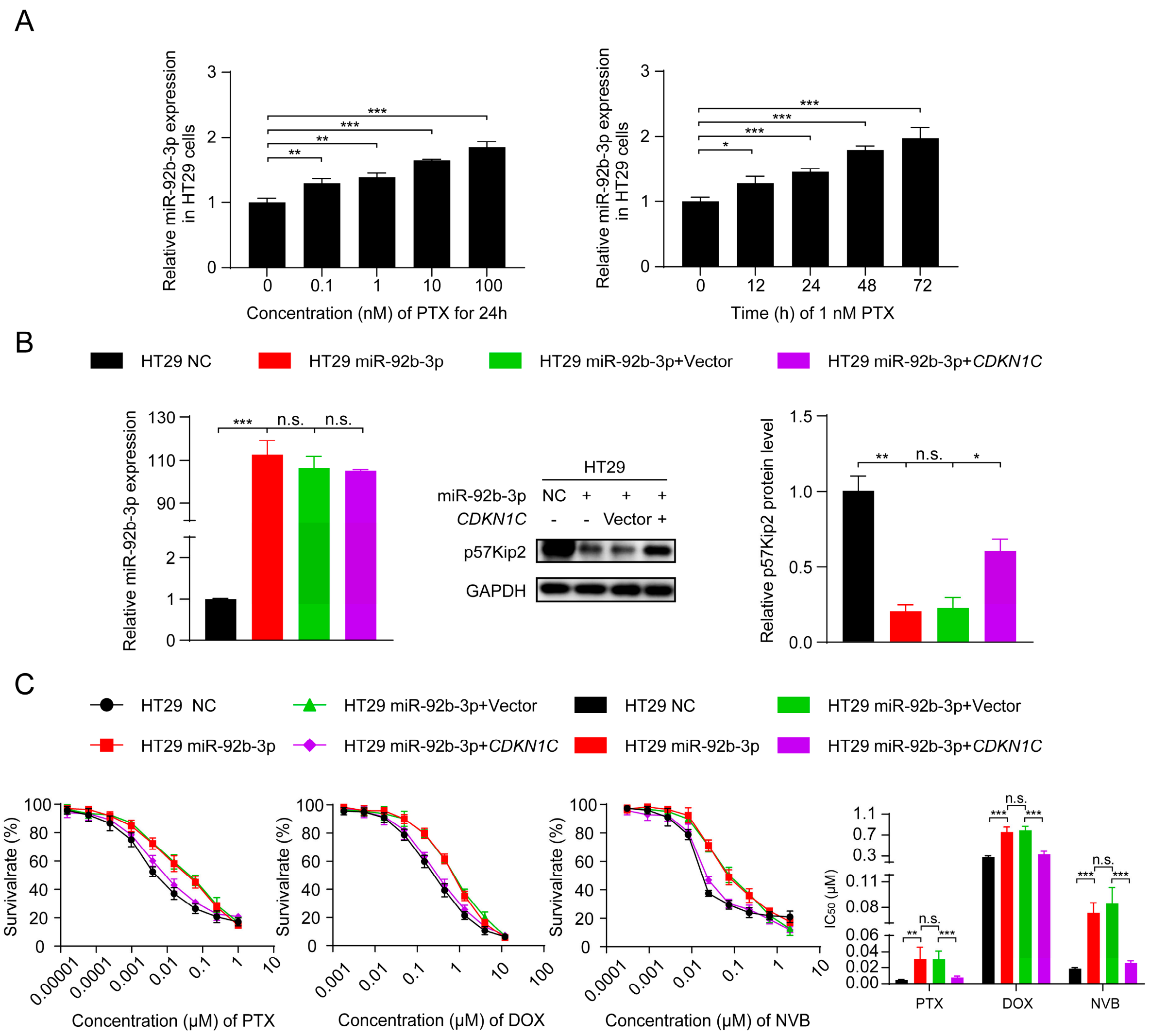

Figure S2. miR-92b-3p regulates the sensitivity of HT29 cells to chemotherapeutic drugs by targeting *CDKN1C*. (**A**) qRT-PCR analysis of relative expression levels of miR-92b-3p in HT29 cells treated with different concentrations of PTX for 24 h and 1 nM PTX for different time. (**B**) Western blotting analysis of the p57Kip2 expression levels in HT29 cells transfected with miR-92b-3p or co-transfected with miR-92b-3p and *CDKN1C*. (**C**) Overexpression of *CDKN1C* attenuates the desensitizes effect of miR-92b-3p on HT29 cells to chemotherapeutic drugs. Cells were treated with drugs at gradient concentrations for 72h after transfection of miR-92b-3p or cotransfection with miR-92b-3p and *CDKN1C* for 24 h, cell survival curves and IC_{50} values of multiple chemotherapeutic drugs were measured by CCK8 assay. All experiments above were repeated three or more times independently and values are shown as mean ± SD. *$p < 0.05$; **$p < 0.01$; ***$p < 0.001$.

Table S1. Primer sequences used for qRT-PCR.

Genes (*Homo sapiens*)	Primers	Sequences (5′-3′)
miR-92b-3p	RT Primer	GTCGTATCCAGTGCAGGGTCCGAGGTATTCGCACTGGATACGACGGAGGC
	Forward	GCGTATTGCACTCGTCCCG
	Reverse	AGTGCAGGGTCCGAGGTATT
U6	RT Primer	GTCGTATCCAGTGCAGGGTCCGAGGTATTCGCACTGGATACGACAAAATA
	Forward	AGAGAAGATTAGCATGGCCCCTG
	Reverse	ATCCAGTGCAGGGTCCGAGG
CDKN1C	Forward	GCGGCGATCAAGAAGCTGT
	Reverse	GCTTGGCGAAGAAATCGGAGA
GAPDH	Forward	GGAGCGAGATCCCTCCAAAAT
	Reverse	GGCTGTTGTCATACTTCTCATGG

Table S2. The sensitivity of HCT8 and HCT8/T cells to chemotherapeutic drugs. Cell survival curves of multiple chemotherapeutic agents were measured by CCK8 assay after 72h treatment with agents (in gradient concentrations) and the IC_{50} values were calculated based on a non-linear regression analysis. The fold-reversal was calculated as the IC_{50} value of HCT8/T cells divided by the IC_{50} value of HCT8 cells. All experiments above were repeated three or more times independently and values are shown as mean ± SD. *versus HCT8 cells. ***$p < 0.001$.

Drugs	IC_{50} (mean ± SD, μM)		Fold Reversal
	HCT8	HCT8/T	
PTX	0.0278 ± 0.0028	6.82 ± 0.30***	245.32
DOX	0.1030 ± 0.0072	2.33 ± 0.11***	22.62
NVB	0.0396 ± 0.0029	1.04 ± 0.07***	26.26
VCR	0.2033 ± 0.0351	2.89 ± 0.17***	14.22
VP-16	2.3560 ± 0.1778	18.96 ± 0.99***	8.05

Table S3. Overexpression of *CDKN1C* reverses the effect of miR-92b-3p overexpression on sensitivity of DLD1 cells to chemotherapeutic drugs. Cell survival curves of multiple chemotherapeutic agents were measured by CCK8 assay after 72h treatment with agents (in gradient concentrations) and the IC_{50} values were calculated based on a non-linear regression analysis. All experiments above were repeated three or more times independently and values are shown as mean ± SD. *versus control cells respectively. **$p < 0.01$; ***$p < 0.001$.

Drugs	IC_{50} (mean ± SD, μM)			
	DLD1 NC	DLD1 miR-92b-3p	DLD1 miR-92b-3p+Vector	DLD1 miR-92b-3p+*CDKN1C*
PTX	0.0159 ± 0.0048	0.0904 ± 0.0226***	0.0957 ± 0.0147	0.0405 ± 0.0046***
DOX	0.2328 ± 0.0167	0.6536 ± 0.0760***	0.7592 ± 0.1374	0.4382 ± 0.0504***
NVB	0.0354 ± 0.0058	0.1326 ± 0.0143***	0.1284 ± 0.0149	0.0622 ± 0.0080***

Table S4. Overexpression of *CDKN1C* reverses the effect of miR-92b-3p overexpression on sensitivity of HT29 cells to chemotherapeutic drugs. Cell survival curves of multiple chemotherapeutic agents were measured by CCK8 assay after 72h treatment with agents (in gradient concentrations) and the IC_{50} values were calculated based on a non-linear regression analysis. All experiments above were repeated three or more times independently and values are shown as mean ± SD. *versus control cells respectively. $^{**}p < 0.01$; $^{***}p < 0.001$.

Drugs	IC_{50} (mean ± SD, μM)			
	**HT29 NC**	**HT29 miR-92b-3p**	**HT29 miR-92b-3p+Vector**	**HT29 miR-92b-3p+*CDKN1C***
PTX	0.0046 ± 0.0048	0.0304 ± 0.0226**	0.0302 ± 0.0147	0.0079 ± 0.0046***
DOX	0.2740 ± 0.0302	0.7573 ± 0.0963***	0.7889 ± 0.0765	0.3315 ± 0.0585***
NVB	0.0187 ± 0.0014	0.0736 ± 0.0119***	0.0846 ± 0.0190	0.0257 ± 0.0027***

Fig.3C

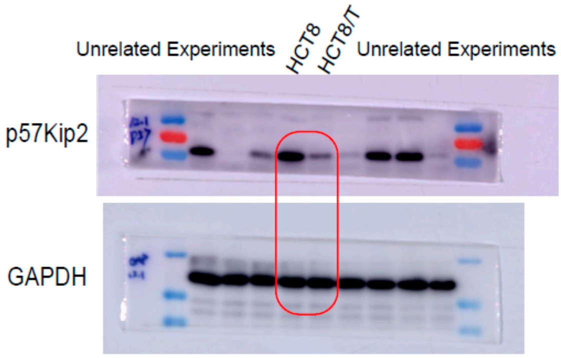

Fig.3E

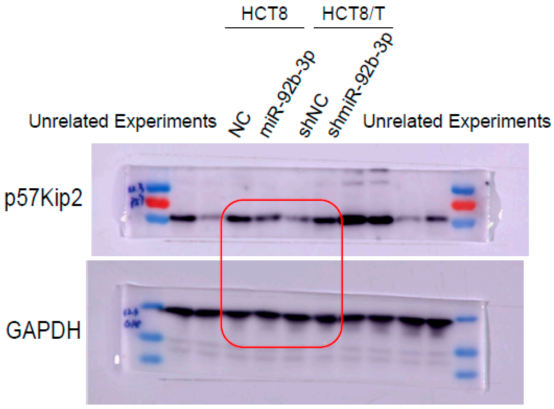

Detail information about Figure 3.

Fig.4A

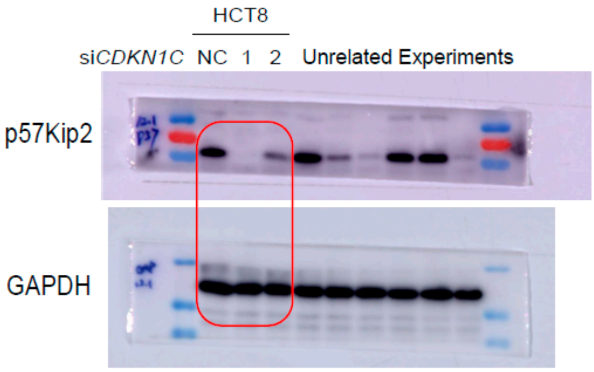

Fig.4C

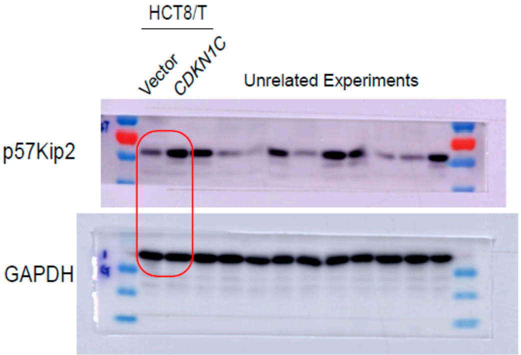

Detail information about Figure 4.

Fig.5A

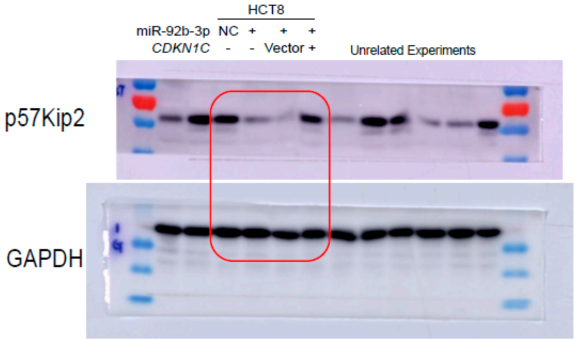

Fig.5C

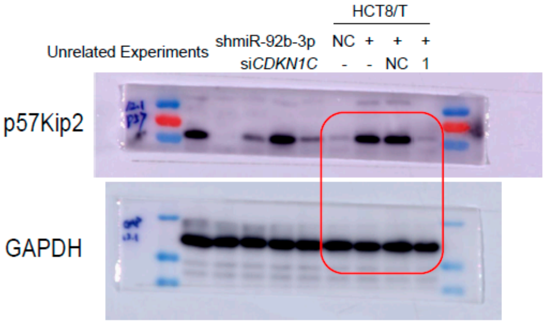

Detail information about Figure 5.

Fig.7H

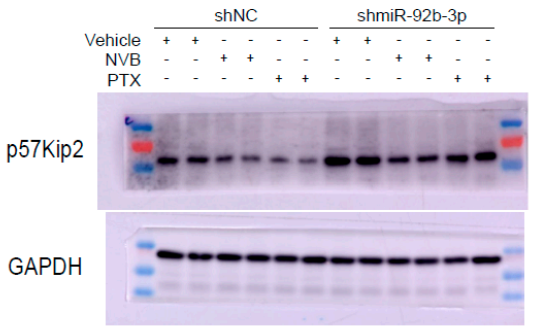

Detail information about Figure 7.

Fig.S1-S2

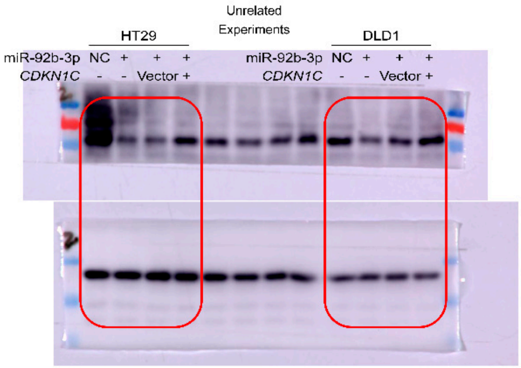

Detail information about Figure S1,S2.
